# Soil Photosynthetic Microbial Communities Mediate Aggregate Stability: Influence of Cropping Systems and Herbicide Use in an Agricultural Soil

**DOI:** 10.3389/fmicb.2019.01319

**Published:** 2019-06-14

**Authors:** Olivier Crouzet, Laurent Consentino, Jean-Pierre Pétraud, Christelle Marrauld, Jean-Pierre Aguer, Sylvie Bureau, Carine Le Bourvellec, Line Touloumet, Annette Bérard

**Affiliations:** ^1^UMR ECOSYS (Ecologie et Ecotoxicologie des Agroécosystèmes), INRA, AgroParisTech, Université Paris-Saclay, Versailles, France; ^2^UMR 6023 LMGE, CNRS, Université Clermont Auvergne, Aubière, France; ^3^UMR 408 SQPOV, INRA, Avignon Université, Avignon, France; ^4^UMR 1114 EMMAH (Environnement Méditerranéen et Modélisation des Agro-Hydrosystèmes), INRA, Avignon Université, Avignon, France

**Keywords:** photosynthetic microbial communities, cyanobacteria, aggregate stability, herbicide, cropping systems, exopolysaccharides

## Abstract

Edaphic cyanobacteria and algae have been extensively studied in dryland soils because they play key roles in the formation of biological soil crusts and the stabilization of soil surfaces. Yet, in temperate agricultural crop soils, little is understood about the functional significance of indigenous photosynthetic microbial communities for various soil processes. This study investigated how indigenous soil algae and cyanobacteria affected topsoil aggregate stability in cereal cropping systems. Topsoil aggregates from conventional and organic cropping systems were incubated in microcosms under dark or photoperiodic conditions with or without a treatment with an herbicide (isoproturon). Physicochemical parameters (bound exopolysaccharides, organic carbon) and microbial parameters (esterase activity, chlorophyll *a* biomass, and pigment profiles) were measured for incubated aggregates. Aggregate stability were analyzed on the basis of aggregate size distribution and the mean weight diameter (MWD) index, resulting from disaggregation tests. Soil photosynthetic microbial biomass (chl *a*) was strongly and positively correlated with aggregate stability indicators. The development of microalgae crusts in photoperiodic conditions induced a strong increase of the largest aggregates (>2 mm), as compared to dark conditions (up to 10.6 fold and 27.1 fold, in soil from organic and conventional cropping systems, respectively). Concomitantly, the MWD significantly increased by 2.4 fold and 4.2 fold, for soil from organic and conventional cropping systems. Soil microalgae may have operated directly via biochemical mechanisms, by producing exopolymeric matrices surrounding soil aggregates (bound exopolysaccharides: 0.39–0.45 μg C g^−1^ soil), and via biophysical mechanisms, where filamentous living microbiota enmeshed soil aggregates. In addition, they may have acted indirectly by stimulating heterotrophic microbial communities, as revealed by the positive effect of microalgal growth on total microbial activity. The herbicide treatment negatively impacted soil microalgal community, resulting in significant decreases of the MWD of the conventional soil aggregates (up to −42% of the value in light treatment). This study underscores that indigenous edaphic algae and cyanobacteria can promote aggregate formation, by forming photosynthetic microbiotic crusts, thus improving the structural stability of topsoil, in temperate croplands. However, the herbicide uses can impair the functional abilities of algal and cyanobacterial communities in agricultural soils.

**Originality/Significance:**

Edaphic algal and cyanobacterial communities are known to form photosynthetic microbial crusts in arid soils, where they drive key ecosystem functions. Although less well characterized, such communities are also transiently abundant in temperate and mesic cropped soils. This microcosm study investigated the communities’ functional significance in topsoil aggregate formation and stabilization in two temperate cropping systems. Overall, our results showed that the development of indigenous microalgal communities under our experimental conditions drove higher structural stability in topsoil aggregates in temperate cropland soils. Also, herbicide use affected photosynthetic microbial communities and consequently impaired soil aggregation.

## Introduction

Photosynthetic microorganisms such as eukaryotic algae and prokaryotic cyanobacteria are ubiquitous pioneer colonizers of topsoil surfaces (Booth, 1941; Metting, 1981). They have been extensively studied in dryland ecosystems, where they play key roles in the formation of biological soil crusts and soil ecological processes (Evans and Johansen, 1999; Belnap and Lange, 2003; Chamizo et al., 2018), and in rice field ecosystems, where they are important to soil fertility (Singh et al., 2011). Far less is known about the communities and functions of soil algae and cyanobacteria living in mesic agricultural croplands (Büdel, 2001; Bérard et al., 2004; Zancan et al., 2006; Langhans et al., 2009; Peng and Bruns, 2019b), located in areas with temperate oceanic climates (Cw, Cfb, or Cfc) or mesic continental climates (Dfa or Dfb) (Peel et al., 2007). Despite their unassuming presence in temperate agricultural soils, algae and cyanobacteria are abundant and diverse (Metting, 1981; Pipe, 1992; Zancan et al., 2006). They can form transient photosynthetic microbiotic soil crusts (Knapen et al., 2007), and they fix N_2_ and CO_2_ (Shimmel and Darley, 1985; Veluci et al., 2006). Thus, like their counterparts in barren arid lands or coastal dunes, they contribute to numerous soil functions. For example, by stabilizing aggregates (Bailey et al., 1973; Metting, 1987) they can protect topsoils against soil erosion (Knapen et al., 2007; Peng and Bruns, 2019b) and limit losses of nutrients and water (Pipe and Shubert, 1984; Langhans et al., 2009; Peng and Bruns, 2019a). Overall, these functions ultimately result in benefits for agricultural soil fertility (Metting, 1990; Renuka et al., 2018).

Soil aggregation plays a key role in protecting soils from water and wind erosion, but it is also involved in other soil functions, such as moisture retention, nutrient retention, and soil carbon sequestration (Le Bissonnais, 1996; Six et al., 2000). Various abiotic and biotic mechanisms operate with different strengths to generate soil aggregates (Tisdall and Oades, 1982). Mineral particles (e.g., clays, silt, metal oxides, alluminosilicates) and cation complexes flocculate together to form small microaggregates (<0.05 mm), which are cemented and bound into larger microaggregates (0.05–0.25 mm) by the biochemical action of soil organic matter and, notably, exopolymeric substances (EPSs) secreted by plant or microorganisms (e.g., exopolysaccharides, Puget et al., 1999; Six et al., 2004). Exopolysaccharides facilitate the adhesion of microbes to aggregates. Then, soil microorganisms, especially those with filamentous phenotypes (e.g., fungal hyphae), become entangled and bind together with microaggregates via biophysical mechanisms, resulting in the formation of macroaggregates (range: 0.25 mm to several millimeters) (Lynch and Bragg, 1985; Chenu and Cosentino, 2011).

The importance of bacteria and fungi in mediating soil aggregate stability has been extensively documented in agricultural soils, research that has underscored the influence of agricultural practices (Chan and Heenan, 1999; Bossuyt et al., 2001; Six et al., 2004). In contrast, almost no research has investigated the contribution of indigenous edaphic algae and cyanobacteria to topsoil aggregation in temperate agricultural soils. Some studies have examined how algae or cyanobacteria promote soil aggregation using inoculation experiments that employed exogenous strains (Bailey et al., 1973; Metting and Rayburn, 1983; Falchini et al., 1996; Peng and Bruns, 2019b). A single field study has described how indigenous algae and cyanobacteria can form microbial crusts that increase the resistance of cropped topsoil to erosion (Knapen et al., 2007). In fact, what we know about the underlying mechanisms by which algae and cyanobacteria influence soil aggregation essentially comes from studies looking at the early stages of biological soil crust formation in arid or semi-arid ecosystems (Malam Issa et al., 2001; Belnap and Lange, 2003). Cyanobacteria and certain algae can bind and cement soil aggregates together, via biophysical (enmeshments with cyanobacterial trichomes) and biochemical (gluing with exuded EPSs) mechanisms (Barclay and Lewin, 1985; Falchini et al., 1996; Malam Issa et al., 2001).

Agricultural practices, such as agrochemical uses, can quickly disrupt microbial communities (Nielsen and Winding, 2002; Imfeld and Vuilleumier, 2012) and can thus impair biological and physicochemical indicators of soil quality and soil functions (Zaady et al., 2013; Rose et al., 2016). Furthermore, as is often the case in microbial ecotoxicology, the aforementioned research has focused almost exclusively on how pesticides impact biomass, abundance, activity, or community composition, and numerous gaps still remain when it comes to assessing the actual impacts on soil functions (Ghiglione et al., 2014). For example, there is limited evidence that agrochemicals affect the soil functions delivered by soil microorganisms (e.g., soil aggregation and erosion) (Bossuyt et al., 2001). Furthermore, while algae and cyanobacteria are known to be affected by cropping systems (Zancan et al., 2006; Zaady et al., 2013) and herbicides (Pipe, 1992; Bérard et al., 2004; Zaady et al., 2004; Crouzet et al., 2013), only one study has shown that agricultural practices (e.g., herbicide uses) may impact the functions of microbial crusts, by disturbing the microalgae component, in semi-arid soils (Zaady et al., 2013).

The research presented here aimed to determine how indigenous photosynthetic microbial communities affected aggregate stability in temperate agricultural soils. Our hypotheses were the following: (i) agricultural practices, such as herbicide use, impair the development of photosynthetic microbial crusts, thereby decreasing soil aggregate stability and (ii) cropping system (conventional vs. organic) shapes microbial communities in such a way that different communities will have different functional roles in soil aggregation and will respond differently to herbicide use (isoproturon was used as a model herbicide). A microcosm approach was employed to simulate the colonization of topsoil aggregates by photosynthetic microbial crusts. We measured different descriptors of the microbial communities making up photosynthetic crusts: chlorophyll *a* concentrations and pigment profiles were used to assess community biomass and structure, and total soil esterase activity served as a proxy for overall heterotrophic activity. Bound exopolysaccharides (i.e., defined in this study as exopolysaccharides bound to soil particles) were extracted and both qualitatively and quantitatively analyzed. Finally, we measured the structural stability of soil aggregates to quantify the significance of microalgal crusts in the aggregation process and assessed the impacts of pesticide use and/or cropping systems.

## Materials and Methods

### Experimental Site

The soil used in the study was sampled at the La Cage Experimental Station (INRA Versailles, France, 48°48′N, 2°08′E). It was taken from plots experiencing one of two cropping systems: a conventional (CONV) cropping system versus an organic (ORG) cropping system. The soil was a silty loam, a loess-derived luvisol (FAO classification system). It had a silty-loam Al horizon (58% silt, 25% sand, and 17% clay) with a neutral pH (6.7–7.1) and a C/N ratio of 9.6–10.3. The cropping systems, in use for the past 20 years, had not significantly altered the main physicochemical variables (pH, organic matter content, cation exchange capacity, levels of major elements such as K, Ca, and Mg) (Autret et al., 2016 and Table 1, reference soil). In these systems, the crop cycle was dominated by short rotations of winter wheat with alfalfa in the ORG system and with rapeseed and peas in the CONV system). No organic fertilizer was applied, and no irrigation was used. Mineral fertilizers (N, P, and K) and pesticides (mostly herbicides and fungicides) were only employed in the CONV system. There was soil tillage in both systems (similar plowing, harrowing, and stubble disking regimes), and additional mechanical weeding was used in the ORG system (Autret et al., 2016).

**Table 1:** Physicochemical properties of soil aggregates from the initial (reference: Ref) soil samples, at day 0 before experiment, and in soil microcosms at the end of incubations (day 50) under the different conditions of incubation: Dark (incubation under dark), Light (incubation with a photoperiod 16/8), Light + IPU (incubation with a photoperiod 16/8 + isoproturon treatment).

	pH	C org (g kg^−1^_dw_)	N tot (g kg^−1^_dw_)	N-NH_4_^+^ (μg g^−1^_dw_)	N-NO_3_^−^ (μg g^−1^_dw_)	Olsen P (μg g^−1^_dw_)
	**soil aggregates from Organic cropping system**					
Ref	7.1 ± 0.2	9.79 ± 0.08	0.95 ± 0.01	38.7 ± 2.8	4.86 ± 0.29	0.087 ± 0.001
Dark	7.1 ± 0.1	9.48 ± 0.12	0.94 ± 0.02	9.8 ± 0.82	3.81 ± 0.34	0.090 ± 0.002
Light	7.3 ± 0.1	10.54 ± 0.24	1.06 ± 0.03	13.5 ± 1.4	3.15 ± 0.44	0.083 ± 0.003
Light + IPU	7.2 ± 0.2	10.19 ± 0.22	1.04 ± 0.02	10.4 ± 1.0	2.99 ± 0.26	0.102 ± 0.003

	**soil aggregates from Conventional cropping system**					
	
Ref	6.6 ± 0.1	9.43 ± 0.06	0.99 ± 0.01	165 ± 13	100 ± 9.0	0.093 ± 0.002
Dark	6.4 ± 0.1	9.36 ± 0.15	0.96 ± 0.02	10.1 ± 0.33	69.6 ± 1.5	0.097 ± 0.003
Light	6.5 ± 0.1	10.01 ± 0.18	1.04 ± 0.04	12.2 ± 0.70	59.6 ± 2.1	0.090 ± 0.003
Light + IPU	6.6 ± 0.1	9.71 ± 0.23	1.02 ± 0.02	9.9 ± 0.47	62.4 ± 1.9	0.106 ± 0.005

*The results were expressed as the mean ± standard deviation (n = 3). C org, organic carbon; N tot, total nitrogen; Olsen P, Olsen available phosphorus.*

### Soil Aggregate Sampling

Soils were sampled in plots under winter wheat cultivation in March 2015, 1 week after an inorganic fertilizer was applied (50 kg N NH_4_NO_3_ ha^−1^) in CONV plots. In both the CONV and ORG plots, the topsoil layer (0–2 cm) was sampled in the interrow zone. Samples were taken from different locations (at least 20 m away from each other and at least 10 m away from plot edges to avoid edge effects) and then mixed to obtain a combined sample for each plot. Soil samples were progressively but not completely air dried in the laboratory and gently crumbled by hand (Le Bissonnais, 1996). Soil aggregates of the target sizes (3.15–5 mm) were obtained by sieving and were stored at 4°C until the microcosm experiment. These were the initial soil aggregates, defined as the reference samples (hereafter Ref, Table 1). The residual aggregates (0–3.15 mm) were also stored.

### Microcosm Experiment

The microcosms were contained in PVC boxes (length: 11.5 cm, width: 9 cm, height: 4.5 cm) equipped with transparent and perforated lids, which allowed air exchange and the illumination of the soil surface. Two days after aggregate preparation, a first soil layer (thickness: 2 cm), composed of the residual aggregates (0–3.15 mm), was placed in the bottom of the boxes; it buffered the microcosm against desiccation. A nylon mesh (Ø: 1 mm) was then added. Upon it was placed a second soil layer (thickness: 1 cm), composed of the Ref soil aggregates (3.15–5 mm). This experiment was intended to reproduce the initial conditions under which soil algae and cyanobacteria colonize soil surfaces, like those we would expect to see in plowed soil.

For each cropping system type, three replicate microcosms were assigned to one of three sets of incubation conditions. In the “dark” treatment, soil aggregates were incubated under continuously dark conditions (i.e., the boxes were wrapped in aluminum foil). In the “light” treatment, soil aggregates were incubated under a 16/8 (light/dark) photoperiod, where PAR (light intensity of 100 μmol m^2^ s^1^) was provided by a 11M1003H RADIOMETRIX^^®^^ LED lighting system [which contains three sets of white (6500K), blue (450 nm), and red (660 nm) LEDs; Alpheus, France]. In the “light + IPU” treatment, soil aggregates were incubated under a 16/8 (light/dark) photoperiod but were sprayed with an herbicide on day 0. The herbicide Matin EL^^®^^ [a commercial formulation of isoproturon (IPU) that contains 500 g IPU L-1; Phyteurop] was used at the recommended field dose (2.4 L ha-1). Distilled water was added to attain 80% mWHC, and the microcosms were incubated for 50 days at 20°C. Soil moisture was maintained once a week. On day 50, the surface layer of soil aggregates (0–1 cm) was carefully sampled and homogenized. Several aliquots were air-dried to carry out the physicochemical, aggregate stability, and bound exopolysaccharide analyses, while others were stored overnight at 4°C to later quantify concentrations of chl *a* and other photosynthetic pigments.

### Soil Physicochemical Characteristics

The analyses of total organic carbon (C_org_), total nitrogen (N_tot_), total inorganic nitrogen (N_min_ = NH_4_^+^ + NO_x_^−^), available phosphorus, and pH_H2O_ of the soil aggregate samples (3.15–5 mm) samples were carried out at the beginning and the end of the experiment. Measurements were performed by INRA’s Soil Analysis Laboratory (Arras, France), in accordance with the ISO normalization procedures. A description of these methods is available on the laboratory’s website^1^.

### Structural Stability of Soil Aggregates

We measured the stability of air-dried aggregates (3.15–5 mm) sampled before incubation (Ref samples, day 0) and after incubation (dark, light, light + IPU treatments, day 50) using the method described by Le Bissonnais (1996). To summarize, the method is based on three disaggregation tests: test 1 employs fast-wetting conditions and addresses slaking mechanisms and the breakdown caused by the compression of the air trapped in aggregate soil micropores during wetting; test 2 employs slow-wetting conditions and examines the differential swelling and shrinking during wetting and drying that results in aggregate microcracking; and test 3 employs mechanical breakdown that mimics the impact of raindrops on wet soil. For each microcosm sample, triplicate subsamples (5 g of dry soil) were analyzed for each disaggregation test. After the disaggregation tests, a combination of wet- and dry-sieving (mesh sizes: 2000, 1000, 500, 250, 100, and 50 μm) was used to determine the resulting distribution of aggregate in seven size classes: >2 mm, 2 – 1 mm, 1–0.5 mm, 0.5–0.25 mm, 0.25–0.1 mm, 0.1–0.05 mm, <0.05 mm. The residual aggregates remaining on each sieve were dried and weighed, and the class-size distribution was determined as a percentage by dry mass of the initial sample. Then, aggregate stability was assessed by the resistance of soil samples against aggregate breakdown. Two indicators resulting from the aggregate-size distribution were used: the percentage of the largest class-size of aggregates (>2 mm) and the mean weight-diameter (MWD) index (Le Bissonnais, 1996). For each test, the mean weight-diameter (MWD) was calculated as follows:

(1) $$\text{MWD}=\sum_{i=n}^{\text{n}}X_{\text{i}}\;p_{\text{i}}$$

where *X*_i_ is the mean diameter of i^th^ mesh size (mm) and *p*_i_ is the proportion of aggregates in the i^th^ fraction. A mean of the MWD between the three tests can be made to summarize the overall response (geometric mean).

### Photosynthetic Microbial Community

The concentration of soil chlorophyll *a* (chl *a*) is an indicator of soil photosynthetic microbial biomass (Tsujimura et al., 2000; Crouzet et al., 2013). Fresh subsamples of soil aggregates (2.5 g) were mixed with 7.5 ml of acetone/water (90v: 10v). The mixture was then shaken for 15 h in the dark at 4°C. The extracted chl *a* were then spectrophotometrically quantified at different wavelengths, and the chl *a* concentrations were calculated using the method and equation described by SCOR-UNESCO Working Group 17 (1966). Other photosynthetic pigments (chlorophyll b, chlorophyll c, fucoxanthin, lutein, diadinoxanthin, neoxanthin, zeaxanthin, and pheophytin a) were quantified using the same acetone extracts and HPLC (in accordance with Zapata et al., 2000 and Joly et al., 2015). This information helped clarify the biochemical structure of the photosynthetic microbial communities. Pigments were identified and quantified via comparisons with analytical standards (DHI Lab Products, Denmark).

### Bound Exopolysaccharides

The fractions of bound exopolysaccharides were extracted from dried soil pellets (see section “Microcosm Experiment”) with cation exchange resin (CER) (Gerbersdorf et al., 2009; Redmile-Gordon et al., 2014) after the preliminary removal of colloidal EPSs via CaCl_2_ extraction. The resulting CER-extracts were then separated into aliquots. Aliquots to be used in the analysis of total bound exopolysaccharides were frozen, and aliquots to be used in the spectrometric analysis and monosaccharide composition analysis were lyophilized.

The total carbohydrates of the bound exopolysaccharides were quantified using the phenol–sulphuric acid method (Dubois et al., 1956). Mid-infrared (MIR) spectra were determined for the bound exopolysaccharide fractions using a Tensor 27 FTIR spectrometer (Bruker Optics, Wissembourg, France) (Bureau et al., 2009). The wavelength range 900–1200 cm^−1^ was used because the intense bands that are specific to polysaccharides occur in this region (Ludwig et al., 2008).

After the MIR analysis, the remaining triplicate lyophilized aliquots were pooled to have sufficient material for the monosaccharide composition analysis. Neutral sugars were analyzed as alditol acetates following acid hydrolysis, in accordance with Renard and Ginies (2009). Uronic acids were measured spectrophotometrically using the m-hydroxydiphenyl assay and galacturonic acid as an external standard.

### Soil Microbial Activity

Fluorescein diacetate (FDA) hydrolysis has been suggested as a suitable indicator of the total heterotrophic activity of soil microbial biomass because many ubiquitous lipases, proteases, and esterases are involved in FDA hydrolysis (Schnürer and Rosswall, 1982). FDA hydrolysis assays were therefore performed using a microplate-based method, in accordance with Green et al. (2006).

### Statistical Analysis

All data were expressed in terms of soil dry weight (dw). Two-way ANOVAs, followed by pairwise *post hoc* tests using Bonferroni corrections (*p* < 0.05), were performed to analyze the effects of the two experimental variables—cropping system (ORG and CONV) and incubation conditions (dark, light, light + IPU)—and their interaction. To meet ANOVA assumptions, the data were checked for normality (Shapiro test) and homoscedasticity (Bartlett test). If the data sets were not normal or homoscedastic, they were transformed (log[x+1] or logit) to meet assumptions. For the ANOVAs, we used linear models (lm), except in the case of the aggregate size classes (% data), for which we used generalized linear models (glm). To investigate differences among the magnitude of responses of a given parameter between two incubation treatments, among the two cropping systems, we used the Mann–Whitney U test.

To assess how the aggregate size distribution or the biochemical structure of microalgal communities were affected by incubation conditions and cropping system, principal component analyses (PCA) were performed on the data of relative abundances of aggregates classes or pigments. MIR-spectral pre-processing and data analysis were performed with Matlab v. 7.5 (Mathworks Inc., Natick, MA, United States); the SAISIR package was employed. Before any data analysis was carried out, standard normal variate (SNV) correction was applied to all the spectra. A hierarchical cluster analysis was performed using Euclidean distances to qualitatively discriminate among the patterns of bound exopolysaccharides for the different incubation conditions. A PERMANOVA was then performed to evaluate the effects of the two experimental variables (cropping system and incubation conditions).

Pearson correlations were used to test the relations among the microbial, biochemical, and physical parameters. Statistical analyses were performed with R software, and a level of statistical significance of α < 0.05 was used.

## Results

### Soil Chemical Properties

The pH of soil aggregates from the conventional (CONV) system was initially lower than that of soil aggregates from the organic (ORG) system; incubation conditions did not affect pH during the experiment (Table 1). Cropping system did not affect the C_org_ and N_tot_ in the Ref samples, but CONV soil aggregates had higher nitrate and ammonium levels than did ORG soil aggregates. At the end of the incubation period, the ORG soil aggregates in the light treatment contained significantly higher C_org_ (0.7–1.0 mg of C g^−1^_dw_) than the ORG aggregates in the dark treatment and the Ref aggregates (Table 1).

### Development and Biomass of Soil Photosynthetic Microbial Crusts

**FIGURE 1:**
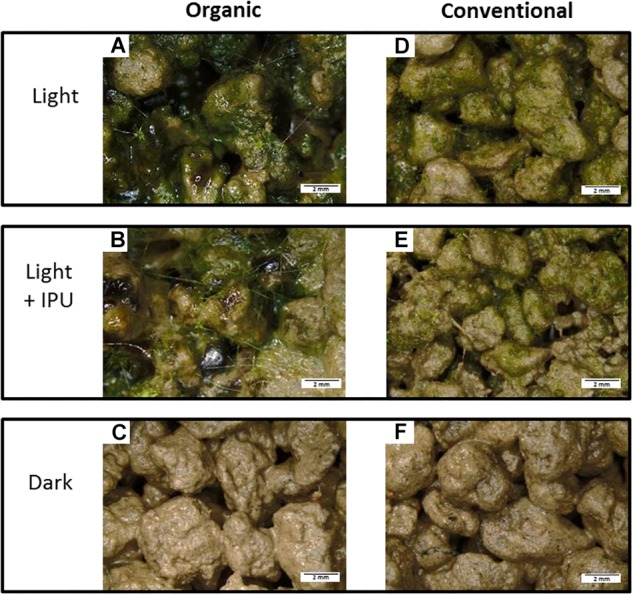
Photosynthetic microbial biofilms growing on soil aggregates from Organic **(A–C)** or Conventional **(D–F)** cropping system, along the different incubation conditions (50 days). The Dark condition **(C,F)** did not show visible algae growth. Various phenotypes of indigenous cyanobacteria and algal colonies covered the aggregate surfaces of incubated microcosms in Light **(A,D)** and Light + IPU conditions **(B,E)** (see Table 1 for acronym description). The filaments bridging between aggregates **(A,B)** are probably cyanobacteria and moss rhizoids.

**FIGURE 2:**
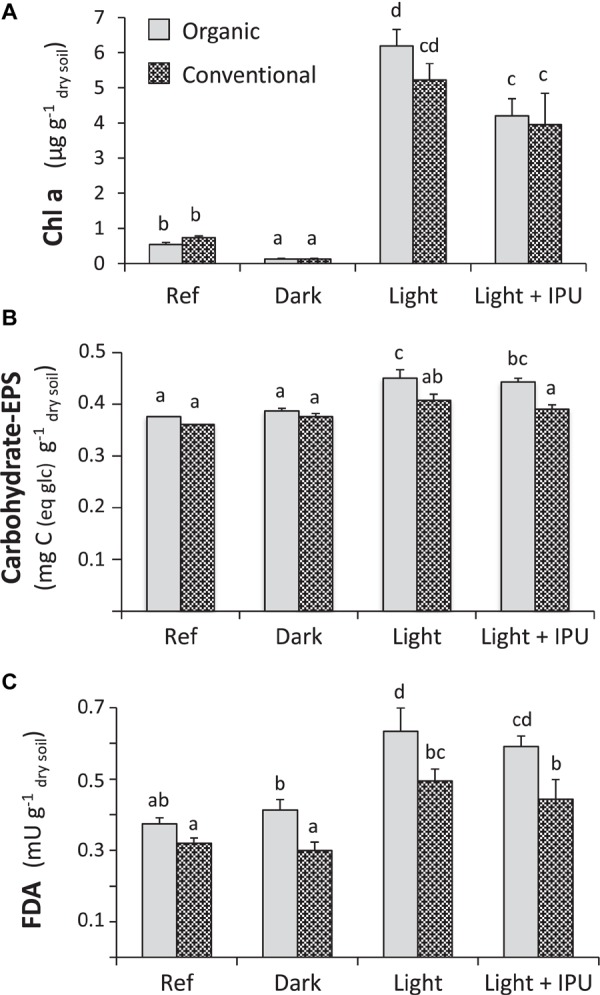
Concentrations of **(A)** Chlorophyll *a* (Chl *a*) and **(B)** bound exopolysaccharides and **(C)** total microbial activity (FDA) of the soil aggregates from Organic and Conventional cropping systems, in the reference soil samples (Ref, at day 0) and in soil microcosms at the end of incubations (day 50) under the different conditions (Dark, Light, Light + IPU, see Table 1 for acronym description). The values are the means with the standard deviation (*n* = 3). Different letters show significant differences between incubation conditions, according a two-way ANOVA (incubation conditions and cropping system as factors) and a Bonferroni corrected *post hoc* comparison (*p* < 0.05).

Ref soil aggregates displayed minimal photosynthetic microbial crusts (visual observation) and low chl *a* concentrations (>1 μg chl *a* g^−1^_dw_). The ANOVA highlighted that incubation conditions had an effect (*F* = 178.3, *p* < 0.001), while cropping system did not (*F* = 2.34, *p* = 0.14); there was no significant interaction (*F* = 2.71, *p* = 0.093). The strong effect of the incubation conditions was mainly due to differences between the dark and light treatments and, to a lesser extent, the presence of the herbicide (light + IPU treatment). In the dark treatment, the photosynthetic microorganisms were not visible (Figure 1C,F), and chl *a* concentrations were so low that they were at the limit of being quantifiable (Figure 2A). In the two light treatments (light and light + IPU), cyanobacteria and algae consistently colonized the surface of soil aggregates (Figure 1A,B,D,E). At the aggregate scale, various phenotypes of photosynthetic microbial crusts (viscous and filamentous) were observed, as was the presence of cyanobacteria and rhizoids of bryophytes (germination stage) (Figure 1A,B). The chl *a* concentrations confirmed the strong development of photosynthetic microorganisms in the light treatment (5.2 and 6.2 μg chl *a* g^−1^_dw_ in aggregates for the ORG and CONV system, respectively) (Figure 2A); this development was less pronounced in the light + IPU treatment (Figure 2A). Compared to the light treatment, the light + IPU treatment induced a significant decrease (−33.2%) in chl *a* concentrations in the ORG soil aggregates (Figure 2A); in contrast the effect (−24.1%) on the CONV soil aggregates was not statistically significant (Figure 2A).

The pigment fingerprint profiles of the soil photosynthetic microbial communities (Supplementary Figure S1) were affected by incubation treatments (light ± IPU; PCA axis 1) and cropping system (PCA axis 2). While the ORG and CONV soil aggregates had dissimilar pigment profiles in the light treatment, they had similar pigment profiles in the light + IPU treatment (Supplementary Figure S1). Concentrations of chl b and fucoxanthin greatly contributed to the differences seen between the light ORG soil aggregates and the light CONV soil aggregates (PCA axis 2), while concentrations of chl *a* and lutein contributed more to the variation due to the application of IPU (PCA axis 1) (Supplementary Figure S1).

### Bound Exopolysaccharides

The Ref and dark soil aggregates from the ORG and CONV systems did not have different concentrations of bound exopolysaccharides (Figure 2B). A two-way ANOVA revealed the strong effects of incubation conditions (*F* = 19.8, *p* < 0.001) and cropping system (*F* = 25.1, *p* < 0.001); there was no significant interaction between the two variables (*F* = 3.02, *p* = 0.08). For soil aggregates from both cropping systems, the light and light + IPU treatments significantly increased concentrations of bound exopolysaccharides, as compared to the dark treatment. No differences were seen between the light and the light + IPU treatments (Figure 2B).

**FIGURE 3:**
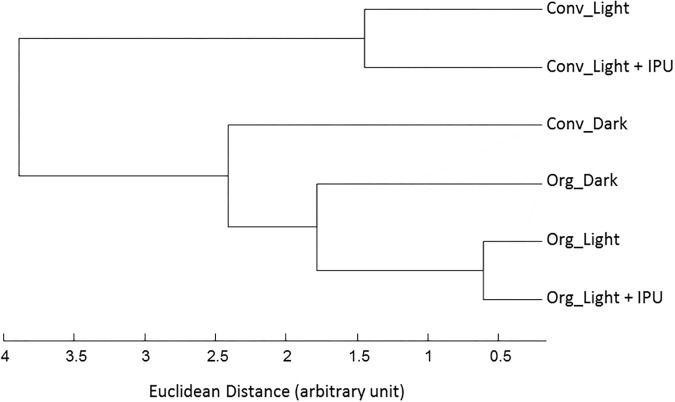
A hierarchical cluster analysis on Mid-Infrared spectra (data ranged from 1500 to 900 cm^−1^) of bound exopolysaccharides samples extracted from soil aggregates of microcosms at the end of incubations (day 50) under the different conditions (Dark, Light, Light + IPU, see Table 1 for acronym description). “Org” is for Organic cropping system and “Conv” is for Conventional cropping systems.

Likewise, the MIR spectra revealed differences in the chemical structures of the bound exopolysaccharides between the dark treatment and the light (±IPU) treatments (based on hierarchical clustering/Euclidean distances). Greater differences were reported among CONV than ORG soil aggregates. The MIR spectra of the bound exopolysaccharides were not statistically different between the light and light + IPU treatments (Figure 3). A PERMANOVA carried out with the spectral data confirmed the significant effect of cropping system (*F* = 100, *p* < 0.001) and revealed the weaker effect of incubation conditions (*F* = 9, *p* < 0.025). The monosaccharide composition analysis of the bound exopolysaccharide extracts suggested that soil aggregates in the light treatment had high levels of monosaccharides, particularly mannose, galactose, glucose, and galacturonic acid, especially in CONV soil aggregates (Supplementary Figure S2); fucose was not detected in soil aggregates in the dark treatment (Supplementary Figure S2). In the light + IPU treatment, only levels of glucose and galacturonic acid were higher in ORG soil aggregates (Supplementary Figure S2).

### Microbial Esterase Activities

There was no significant difference in overall microbial activity in Ref versus dark soil aggregates, regardless of cropping system (Figure 2C). A two-way ANOVA showed that incubation conditions (*F* = 51.2, *p* < 0.001) and cropping system (*F* = 50.1, *p* < 0.001) had significant effects on overall microbial activity; there was no significant interaction (*F* = 1.7; *p* = 0.206). At the end of the incubation period, microbial activity was significantly higher in ORG versus CONV soil aggregates, regardless of treatment group. The main differences occurred between the soil aggregates in light versus dark treatments: the microbial activity values were 62.2 and 70.1% higher in the light than in the dark treatment for the ORG and CONV soil aggregates, respectively (Figure 2C). The light + IPU treatment did not affect overall microbial activity (Figure 2C).

### Aggregate Size Distribution and Aggregate Stability

**FIGURE 4:**
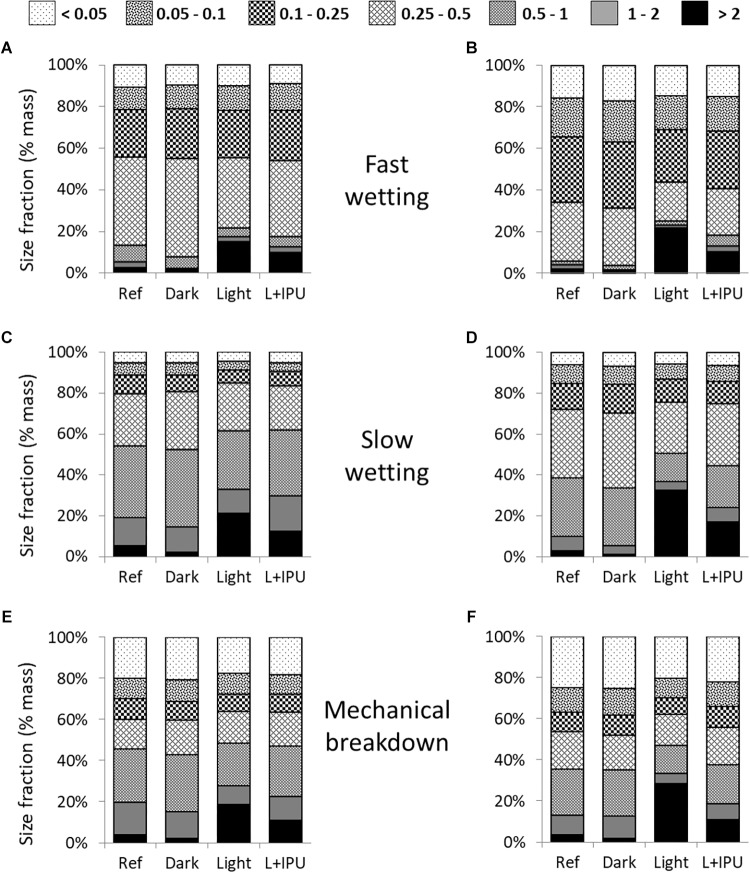
Relative distribution of the aggregate size fractions determined following three aggregate stability tests, for the reference soil aggregates (Ref, at day 0) and soil aggregates from Organic **(A,C,E)** or Conventional **(B,D,F)** cropping systems (*n* = 3), at the end of microcosm incubations (day 50) under the different conditions (Dark, Light, Light + IPU, see Table 1 for acronym description). For a better graphical understanding, the standard deviations and statistical analyses were only shown for the largest aggregates (>2 mm) in Table 2.

The aggregate size distributions resulting from each stability test are depicted in Figure 4. Among the three tests, the fast-wetting test appeared to result in the most destructive disaggregation, with the lowest MWD indexes for ORG and CONV soil aggregates. In opposite, the slow-wetting test seemed to be the least destructive test.

**FIGURE 5:**
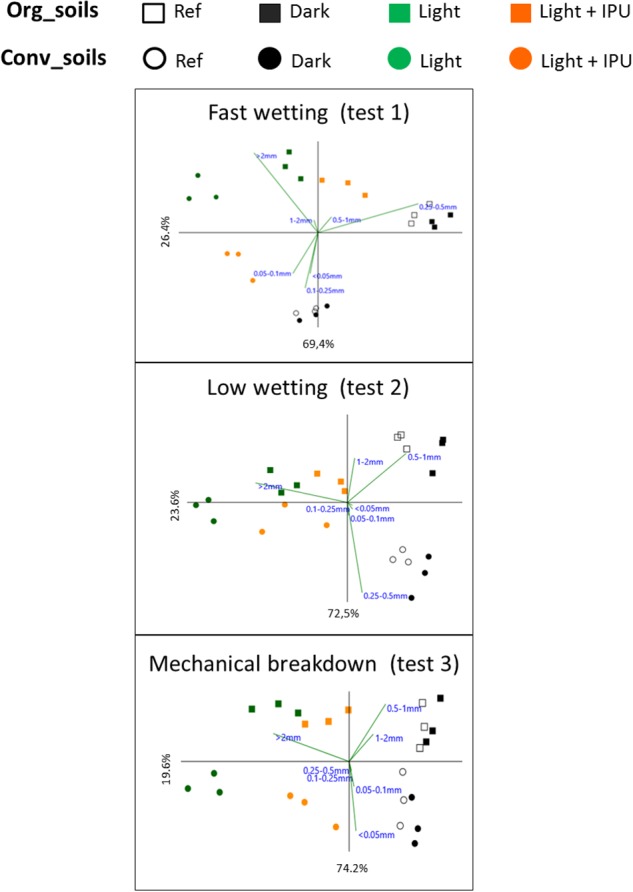
Principal component analyses of the class size distribution, following each stability tests, of the soil aggregates from Organic (filled squares) or Conventional (filled circles) cropping systems, at the end of microcosm incubations (day 50) under the different conditions (Dark, Light, Light + IPU, see Table 1 for acronym description). Reference soil aggregates (Ref, at day 0) are shown by open symbols. The biplot (vectors lengths) shows the contribution of each class size to the difference among groups.

With regard to the total aggregate size distribution, the PCA analyses (Figure 5) underscored the differences due to incubation conditions (PCA axis 1) and cropping systems (PCA axis 2). These effects were statistically confirmed by a two-way PERMANOVA (Supplementary Table S1). PCA1 suggested that the effect of the incubation conditions was principally due to differences between the light and dark treatments and that there was an intermediate effect associated with the light + IPU treatment, especially in the case of the CONV soil aggregates (Figure 5). The effects of photoperiodic incubation (versus dark treatment) mainly manifested themselves in significant shifts in macroaggregate percentages from the smaller class-sizes (1–2 mm; 0.5–1 mm; 0.25–0.5 mm) to the largest class-size (>2 mm) (Figure 4 and Table 2). As example, in light treatments, the amounts of large aggregates (>2 mm) has reached up to 21.5 and 32.5 % of the total dry mass of aggregates, in organic and conventional cropping systems, respectively, following the slow-wetting test (Table 2). In dark treatments, these values remained very low at 2.3 and 1.3 % of the total dry mass of aggregates, in organic and conventional cropping systems, respectively (Table 2). The magnitudes of these shifts between dark and light treatments were significantly greater for the CONV soil aggregates (by 22.7 fold in the fast-wetting test, 27.1 fold in the slow-wetting test, 14.9 fold in the mechanical-breakdown test) than for the ORG soil aggregates (by 10.6 fold in the fast-wetting test, 9.4 fold in the slow-wetting test, 8.4 fold in the mechanical-breakdown test; following data listed in Table 2, Mann–Whitney U test, *p* < 0.01). Concomitantly, for all three tests, the percentages of microaggregates (sum of class sizes <0.25 mm) in the CONV soil decreased significantly for the light treatments (±IPU) but not for the dark treatment (Figure 4, two-way ANOVA *post hoc* test). Overall, the MWD (considering all the aggregate size distribution) of CONV soils significantly increased in the light treatments, as compared to dark or reference soil samples, following all three tests (Table 2). The magnitude of the increase of the MWDs, in light compared to dark incubations, was significantly greater for the CONV soil aggregates (from 0.22 to 0.92 corresponding to an increase by 4.2 fold) than for the ORG soil aggregates (from 0.31 to 0.75 corresponding to an increase by 2.4 fold), in the fast-wetting test (resistance to slaking; Mann–Whitney U test, *p* < 0.01). In the other tests, the increase were 1.8 fold in the slow-wetting test and the mechanical-breakdown test, for the ORG soil aggregate and 2.9 fold in the slow-wetting test and 2.6 fold the mechanical-breakdown for the CONV soil aggregates (following data listed in Table 2, Mann–Whitney U test, *p* < 0.05).

**Table 2:** Percentages of the largest aggregates (>2 mm) and mean weight diameter (MWD in mm) indexes generated following each stability test performed on the incubated soil aggregates from Organic and Conventional cropping systems.

		Test 1 Fast-wetting		Test 2 Slow-wetting		Test 3 Mechanical breakdown	
		[>2 mm]	MWD	[>2 mm]	MWD	[>2 mm]	MWD
**Organic soil aggregates**	Ref	2.5 ± 0.7 b	0.38 ± 0.03 a	5.4 ± 0.8 b	0.77 ± 0.04 b	3.9 ± 0.7 a	0.64 ± 0.03 ab
	Dark	1.4 ± 0.3 a	0.31 ± 0.02 a	2.3 ± 0.7 a	0.68 ± 0.03 b	2.2 ± 0.2 a	0.56 ± 0.04 ab
	Light	14.9 ± 3.0 b	0.75 ± 0.09 bc	21.5 ± 4.8 d	1.24 ± 0.11 cd	18.6 ± 3.3 c	1.02 ± 0.09 de
	Light + IPU	9.6 ± 2.9 b	0.59 ± 0.08 b	12.5 ± 2.4 c	1.03 ± 0.09 c	10.9 ± 2.0 b	0.82 ± 0.07 cd
**Conventional soil aggregates**	Ref	1.9 ± 0.5 a	0.28 ± 0.01 a	3.1 ± 0.5 a	0.57 ± 0.02 ab	3.7 ± 0.5 a	0.53 ± 0.03 ab
	Dark	1.0 ± 0.3 a	0.22 ± 0.02 a	1.2 ± 0.4 a	0.48 ± 0.03 a	1.9 ± 0.5 a	0.49 ± 0.03 a
	Light	21.7 ± 1.5 c	0.92 ± 0.06 c	32.5 ± 4.1 e	1.40 ± 0.11 d	28.3 ± 4.0 d	1.25 ± 0.11 e
	Light + IPU	10.1 ± 3.1 b	0.58 ± 0.11 b	16.9 ± 4.6 cd	0.99 ± 0.13 c	11.0 ± 4.1 bc	0.73 ± 0.12 bc
**Two-way ANOVA statistics**	Treatment	<0.001	<0.001	<0.001	<0.001	<0.001	<0.001
	Crop_syst	0.036	0.738	0.004	0.040	0.582	0.682
	interaction	0.007	0.007	0.002	0.001	0.014	0.007

*Reference soil samples (Ref) are the initial soil aggregates at day 0, before incubation). Dark, Light and Light + IPU are soil aggregates at the end (day 50) of the different incubation conditions (see Table 1 for acronym description). The values are the means with the standard deviation (n = 3). For each disaggregation test, different letters indicated significant differences between incubation conditions (two-way ANOVA, with treatment and cropping systems as factors, followed by Tukey’s post hoc test, p < 0.05). See Figure 4 for the overall class size distributions.*

Furthermore, along the PCA2 axis (Figure 5), there were large differences in the overall size-class distribution between the ORG and CONV soil aggregates from the Ref samples and the dark treatment; however, these differences were lessened for the soil aggregates in the light treatments, notably following the slow-wetting test. More specifically, for all three tests, higher percentages of the largest aggregate (>2 mm) were recovered from CONV than ORG soil aggregates from the light treatments (Table 2). Nevertheless, for a given set of incubation conditions, greater percentages of total macroaggregates (sum of class sizes >0.25 mm) were generated from ORG versus CONV soil aggregates following the three tests; the only exception was seen for aggregates from the light treatments following the mechanical-breakdown test (Figure 4, two-way ANOVA *post hoc* test). Overall, the MWD values did not reveal any differences in structural stability between the ORG and CONV soils for a given set of incubation conditions.

When the effects of herbicide use were examined, the PCAs displayed the aggregates from the light + IPU treatments in an intermediate position between those from the light and dark treatments, whatever the cropping system, (Figure 5). The largest aggregates (>2 mm) were the most responsive class of aggregates. It showed significant decreases in the light + IPU, as compared to the light treatments, after all three disaggregation tests, with the exception of the ORG soil aggregates after the fast-wetting test (Table 2). The highest decreases were recorded following the mechanical breakdown test for CONV soil (from 28.3 to 11 %) and in a similar way following the slow-wetting test (from 21.5 to 12.5 %) or the mechanical breakdown test (from 18.6 to 10.9 %) (Table 2). There were concomitantly higher amounts of intermediate macroaggregate sizes (1–2 mm; 0.5–1 mm; 0.25–0.5 mm) in the light + IPU treatment, except for the CONV soil aggregates following the mechanical-breakdown test (Figure 4). In this laster case, there was a significant decrease in the percentage of microaggregates (<0.25 mm) in the light + IPU treatment, compared to the light treatment (Figure 4; two-way ANOVA *post hoc* test, *p* < 0.01). Overall, the MWD of the CONV soil aggregates was significantly impaired by the light + IPU treatment compared to the light treatment (Table 2). The decreases were −38, −29, and −41%, following the fast-wetting, slow-wetting, and mechanical-breakdown tests, respectively. No significant effect occurred in the MWD index of the ORG topsoil aggregates (Table 2).

### Relations Among Biotic and Abiotic Aggregate Properties

The chl *a* concentrations were highly correlated with the FDA hydrolysis values, the bound exopolysaccharide concentrations, and C_org_ (Table 3 and Supplementary Figure S3). The percentage of the largest fragments (>2 mm; mean for the three tests), considered to be a proxy of aggregate stability, was strongly correlated with the chl *a* concentrations, C_org_, and the FDA hydrolysis values; it was also correlated to a lesser degree with the bound exopolysaccharide concentrations. Correlations between another proxy of aggregate stability, mean MWD (mean for the three tests), and the biochemical parameters (chl *a* concentrations, FDA hydrolysis values, bound exopolysaccharide concentrations, and C_org_) were significantly different between the CONV and ORG soil aggregates (Supplementary Figure S3).

**Table 3:** Spearman correlation coefficients (bottom left) and *p*-values (top right), among microalgae, microbial and aggregates parameters, including the whole data set of Conventional and Organic soil aggregates.

	chl *a*	EPS	C_org_	FDA	>2 mm
chl *a*		<0.001	<0.001	<0.001	<0.001
Bound-EPS	0.682		<0.001	<0.001	<0.001
C_org_	0.787	0.803		<0.001	<0.001
FDA	0753	0.871	0.823		<0.001
>2 mm	0.932	0.698	0.803	0.777	

*Bound-EPS: total bound exopolysaccharides; >2 mm: geometric mean of the largest aggregates between the three tests. Graphics were shown in Supplementary Figure S3.*

## Discussion

### The Development of Indigenous Photosynthetic Microbial Crusts Induces Soil Aggregation

This microcosm experiment showed that there was a strong development (4–6 μg chl *a* g^−1^_dw_) of indigenous algal and cyanobacterial crusts on soil surfaces under optimal and stable laboratory conditions. Chlorophyll *a* concentrations, which are a proxy for photosynthetic microbial biomass, fell in the same range as those seen in previous laboratory incubation experiments (Crouzet et al., 2013; Joly et al., 2015) and in some field studies (Tsujimura et al., 2000; Lin et al., 2013). The low values observed in the reference samples were typical for field soil samples at the end of winter (early March).

As expected, aggregate stability was greatly enhanced by the growth of soil microalgae in the two light treatments (light ± IPU). The mechanism primarily appeared to be the formation of large water-stable aggregates. Consequently, the increase in the percentage of the largest aggregates (>2 mm) resulted from greater cohesion among smaller macroaggregates (ranging from 0.25 to 2 mm) whose the proportion decreased. Similar patterns of aggregate size distribution were observed when semi-arid soil was inoculated with cyanobacteria (*Nostoc* spp.), even if the strong increases in the large macroaggregates were related to higher decreases of the microaggregates (>0.25 mm) than in our work (Malam Issa et al., 2007). Overall, in accordance with the hierarchal model of soil aggregation (Tisdall and Oades, 1982), each level of micro- and macroaggregation was stabilized by materials and mechanisms of a different nature. The biochemical action of soil organic matter via flocculation and cementation usually takes place as a result of internal mechanisms in microaggregates over the long term. In contrast, the biophysical action of soil organisms impacts the overall external cohesion of macroaggregates over the short term. As a result, it is likely that both the processes of macroaggregation share with the microorganisms a high level of responsiveness to environmental changes (Degens, 1997; Six et al., 2004). Consequently, given that this experiment occurred over the short term, the pronounced increase and responsiveness of the large macroaggregate fraction suggests that, in our work, the microalgae affected aggregation mainly by enhancing physical mechanisms. Most commonly, thick networks of algae, cyanobacterial trichomes (colonial filamentous forms), and other stimulated microbial components (e.g., fungal hyphae) become enmeshed with soil particles and existing aggregates to form yet larger aggregates. Complementary biochemical mechanisms resulting from the production of exopolymeric substances (EPSs: polysaccharides, proteins, amino acids, certain lipids, nucleic acids) produced by soil microorganisms may also be involved (Tisdall and Oades, 1982; Six et al., 2004). In this study, increases were observed in the concentrations of bound exopolysaccharides and, to a lesser extent, in C_org_; they were strongly related to the development of microalgal biomass and increases in indicators of aggregate stability (aggregates >2 mm and MWD). In long-term greenhouse or field experiments, inoculation with algae and cyanobacteria has increased the polysaccharide contents of irrigated agricultural soils and contributed to higher aggregate stability (Metting and Rayburn, 1983; Metting, 1987; Falchini et al., 1996) and proportions of large aggregates Malam Issa et al., 2007). Many cyanobacteria and other non-filamentous microalgae secrete large amounts of various exopolymeric substances, including numerous exopolysaccharides, which form a mechanical structure covering aggregates that reinforces biophysical cohesion (Mazor et al., 1996). Notably, cyanobacterial trichomes are surrounded by mucilaginous sheaths, which enable them to strongly adhere to each other and to soil aggregates, resulting in a gluing mesh (Falchini et al., 1996; Malam Issa et al., 2007). Exopolysaccharides can also be released into the surrounding soil (Redmile-Gordon et al., 2014). In our study, since the microaggregates (<0.25 mm) were not drastically affected in either the light or dark treatments (i.e., whether or not microalgae were present), it is likely that the exopolysaccharides produced by the microalgae mainly acted at the macroaggregate scale via external cohesion, coating aggregate surfaces and gluing cells onto soil particles. This biochemical mechanism thus reinforced the biophysical action of these microbial crusts and mainly operated on macroaggregation. In an incubation experiment, the resistance of inoculated soil aggregates to breakdown is likely related to the changes in micromorphological characteristics of the microbiotic crust, induced by cyanobacterial filaments and EPS (Malam Issa et al., 2007).

It is not just the quantity of bound exopolysaccharides that matters. Their chemical composition may also affect aggregate stability because different bound exopolysaccharides have different binding strengths and hydrophobic properties (Puget et al., 1999; Hu et al., 2003; Rossi and De Philippis, 2015). As a result, the differences in the biochemical quality of bound EPSs (as reflected in the MIR spectra) in aggregates from the light versus dark treatment—differences due to presence or absence of algae—could have contributed to differences in aggregate stability. Following the fast-wetting test (and the mechanical-breakdown test), there was a decrease in the percentage of microaggregates when microalgae were present versus absent (including in the reference aggregates). This pattern may have occurred because there were larger amounts of hydrophobic materials coating the aggregate surfaces, thus slowing down aggregate wetting and decreasing slaking. Interestingly, fucose—a hydrophobic deoxy-hexose known to increase cohesion among soil particles (Chen et al., 2014; Rossi and De Philippis, 2015)—was present in aggregates from the light (±IPU) treatments but absent in aggregates from the dark treatment. Likewise, galacturonic acid, which is suspected to play an important role in the great affinity of cyanobacteria (*Microcoleus vaginatus*) for soil particles (Hu et al., 2003), was present at higher concentrations in aggregates from the light (±IPU) treatments than in aggregates from the dark treatment. However, concentrations of other sugars known to be highly hydrophobic, such as arabinose and rhamnose (Hu et al., 2003; Rossi and De Philippis, 2015), did not differ in the presence or absence of microalgae (light vs. dark treatment). These are some of the first results to address the biochemical quality of microbial exopolysaccharides in soils (e.g., Rossi and De Philippis, 2015 in arid soils). Consequently, further analysis is needed to better understand their role in the aggregation of soil particles.

Furthermore, the development of indigenous soil microalgal crusts promoted overall microbial activity (FDA hydrolysis values). Similar results have previously been obtained in soils inoculated with exogenous algal or cyanobacterial strains (Rogers and Burns, 1994; Acea et al., 2003; Nisha et al., 2007). Such stimulation of heterotrophic microbial components could have been induced by the release of extracellular polysaccharides used as readily available carbon sources (Mager and Thomas, 2011). The development of indigenous microalgal communities drives the formation of complex microbial hot spots on the soil surface (photosynthetic microbiotic crusts, Evans and Johansen, 1999). Bacterial and fungal exopolymeric substances and by-products as well as filamentous forms (i.e., hyphae) are known to play a major role in biochemical and physical aggregation processes (Lynch and Bragg, 1985; Six et al., 2004). They may have amplified the microalgae-related effects on soil aggregation. Overall, microalgae productivity was responsible for the increase in C_org_, as previously observed in an experiment involving algae inoculation (Nisha et al., 2007). Consequently, soil microalgae clearly carry out ecological, physical, and chemical engineering in agricultural soils, as previously demonstrated in degraded temperate soils (Rogers and Burns, 1994; Acea et al., 2003) and arid soils (Evans and Johansen, 1999).

### Impact of Herbicide Use on Algae-Mediated Soil Aggregation

Based on the results for the light versus the light + IPU treatments, the decrease of chlorophyll *a* concentrations showed that the herbicide IPU partially inhibited the microalgae component of the photosynthetic microbial crusts, especially in soil aggregates from the organic cropping system. Several works have already shown the harmful effects of herbicides on the soil microalgae abundances (Metting and Rayburn, 1979; Pipe, 1992), *chl* a biomasses (Zaady et al., 2004, 2013; Crouzet et al., 2013) and photosynthetic activity (Bérard et al., 2004). The pigment fingerprint profiles were also modified by the herbicide application, which may indicate that there were shifts in microalgal community composition. Microalgae species have different pigments, and the pigment composition of a given species may vary according to its physiological state, especially if herbicides induce conditions of stress (Bérard and Pelte, 1999). That said, pigment profiling of soil or water samples remains a suitable and widely used approach for describing phytoplankton community structure (Zapata et al., 2000) and for examining how microalgal community composition responds to herbicides (Dorigo et al., 2004; Joly et al., 2015). There are few studies that have looked at the effects of phenyl-urea herbicides on soil microalgal biomass and community composition (Pipe, 1992). Here, we used agriculturally relevant field doses of one such herbicide, IPU, and confirmed its negative impacts on soil microalgae biomass and changes in biochemical community structure. Also, the application of IPU resulted in a convergence of microalgal communities for aggregates from the two cropping systems, which displayed greater differences in the light treatment. Since lutein and chlorophyll *a* and *b* were the most affected, it seems that IPU might affect *Chlorophyceae* more than other microalgae groups.

As previously discussed, in the light treatment, there was a microalgae-mediated effect on aggregate stability. Consequently, it was expected that IPU’s disturbance of algal and cyanobacterial communities would modify their functional contribution to soil aggregation. In an experiment using high doses of a fungicide, the impairment of fungal biomass functionally disrupted macroaggregate formation (Bossuyt et al., 2001). In our study, the IPU treatment at the beginning of the incubation impaired the percentages of large aggregates (>2 mm) and the MWD values, highlighting that the herbicide impaired the soil aggregate stability, albeit mostly in conventional cropping systems. The previous discussions on the relations between microalgae and aggregation tackled the role of exopolysaccharides. IPU impacted the growth of green algae (*Chlorella* sp.) and cyanobacteria (*Anabaena* sp.) as well as their production of carbohydrates (Mostafa and Helling, 2002). At the community level, the harmful effects of an herbicide (simazine) on both the photosynthetic microbial biomass and the soil polysaccharide contents have been evidenced in semi-arid soils (Zaady et al., 2004, 2013). Here, however, the field dose of the herbicide IPU did not have a significant effect on the levels of bound exopolysaccharides. It is possible that IPU-tolerant microalgae were present and increased their exopolysaccharide production in response to the toxic stress. Such a phenomenon has been previously described for periphyton communities exposed to toxicants (Serra and Guasch, 2009). Also, because we did not know the proportions of the bound exopolysaccharides produced by the microalgae, as well as by the bacteria and fungi, whose presence was stimulated by microalgal growth, their respective contribution to the bound exopolysaccharides remained unclear. Consequently, the concentrations of bound exopolysaccharides failed to explain the effect of IPU on the link between microalgal growth and aggregate stability. It is possible instead that the application of IPU led to lower aggregate stability because soil aggregates were less physically covered with enmeshed microalgal filaments, as a direct result of the decrease in algal and cyanobacterial biomass. To test this hypothesis, further research is needed to explore biofilm structure at the microscopic scale and the phenotypic traits of sensitive algal and cyanobacterial strains.

Overall, such an impact on topsoil aggregation may have drastic implications for soil fertility and soil erosion. In field studies, Zaady et al. (2004, 2013) have shown that the herbicide simazine, by inhibiting the microalgae component of microbiotic crusts, increased soil erosion and C_org_ or nitrate losses, in a semi-arid soil. In our work, negative relationships have evidenced between microalgae biomass, indicators of aggregate stability and exopolysaccharides or C_org_ contents. However, these correlations were strongly shaped by the differences between dark *vs.* light treatments, albeit the data of light + IPU often displayed an intermediate position.

### Effect of Soil Cropping System on Algae-Mediated Aggregation

After 50 days in the light treatment, soil aggregates from the conventional and organic cropping systems displayed no difference in their total chlorophyll *a* concentrations. That said, their pigment profiles were not the same. However, the pigment profiles were not consistent enough to allow the clear identification of the microalgae groups that were potentially dominant in each cropping system. Agricultural practices (i.e., pesticide and fertilizer use), which differ between organic and conventional systems, have been shown to influence the taxonomic composition of algal and cyanobacterial communities in cropping systems (Pipe, 1992; Zancan et al., 2006; Lentendu et al., 2014). Even if our experiment found no effect of cropping system on total photosynthetic biomass, differences in community composition can lead to different functional outputs or differences in community sensitivity to disturbance.

The soil aggregates from conventional and organic systems responded differently to the fast-wetting test, which employed slaking. The results underscore that initial aggregate stability and the functional effects of microalgal growth also differed. In fact, soil aggregates from the conventional system had a lower initial percentage of largest macroaggregates (>2 mm) and a higher percentage of microaggregates, which could suggest they were initially less stable than those from the organic system (likely due to the long-term effects of agricultural practices, Chan and Heenan, 1999; Elmholt et al., 2000). In the light treatments (±IPU), the percentage of the largest macroaggregates (>2 mm) increased dramatically and the percentage of the microaggregates declined strongly in the conventional soil, suggesting that microalgae-mediated aggregation had been more effective than in the organic soil. More specifically, as revealed by the fast-wetting test, microaggregates (<0.25 mm) seemed less prone to form macroaggregates via microalgae-mediated effect in soils from the organic cropping system (i.e., there was no difference in the percentage of microaggregates between the light and dark treatments) versus in soils from the conventional cropping system. It is likely that such differences in MWD were not observed between the two cropping systems because, for the calculation of the MWD values, there was compensation by the smaller macroaggregate size classes (1–2 mm, 0.5–1 mm and 0.25–0.5 mm) in the organic soil. However, these greater benefits of microalgae for the largest aggregates of the conventional soil were not explained by higher levels of chlorophyll *a*, bound exopolysaccharides, C_org_, or microbial activity; indeed, these variables had higher values in organic soil aggregates. It is unlikely that differences in the structural composition of the bound exopolysaccharides were involved in these aggregation patterns because, such differences usually play a greater role in microaggregation—by gluing soil particles together. Moreover, the differences in the correlations observed between the biotic and abiotic variables for the soil aggregates from the two systems (Supplementary Figure S3) suggest that different biological and physical interactions may have been involved in aggregation dynamics and that the nature of these interactions may depend on legacy effects of agricultural practices.

Finally, the application of IPU had a significantly smaller effect on photosynthetic microbial biomass in conventional versus organic soil aggregates. A field study comparing uncultivated soils and cultivated soils subject to many years of pesticide treatments found that soil algal and cyanobacterial isolates from uncultivated soils were less tolerant to di-allate and MCPA herbicides than isolates from adjacent cultivated soils (Metting and Rayburn, 1979). The PICT (pollution-induced community tolerance) concept suggests that a causal relationship exists between a field’s exposure to a toxicant and the sensitivity of its microbial community to the same toxicants (Bérard et al., 2004). In the conventional system from which we took our soil samples, pesticide formulations containing IPU have been applied every 2 years for the past 20 years. As a result, it is likely that IPU-tolerant populations of algae and cyanobacteria have been selected for, which means that the overall community was likely more tolerant of the presence of IPU in the microcosm experiment. For example, a tolerance mechanism favored by some algae or cyanobacteria can be the degradation of the IPU (Mostafa and Helling, 2001).

In contrast, the functional response of the microalgae that contributed to soil aggregation was significantly more affected by IPU in the conventional versus the organic soil aggregates: the systematic decrease in MWD values in the light + IPU treatment was only seen for the aggregates from the conventional system. One hypothesis may be that microalgae incur fitness costs when acquiring tolerance to herbicide-induced stress. In other words, higher energy demands are placed on microorganisms that are coping with the toxicity of IPU (Bérard et al., 2014). As a result, the functional competence of microalgal communities (e.g., aggregation) could be reduced when they are faced with chemical stressors.

## Conclusion

Indigenous cyanobacterial and algal communities that form photosynthetic microbiotic crusts in agricultural cropped soils should be viewed as engineering microorganisms. Indeed, they contribute to aggregate formation and stabilization and thus help protect the soil surface of cropland. The growth of indigenous microalgae primarily favors the formation of large macroaggregates, as pre-existing small aggregates are physically enmeshed by networks of filamentous microbial biomass. Also, microalgal mats coat the surface of macroaggregates (i.e., with biomass and EPS matrices), thus protecting aggregates against slaking via hydrophobic interactions. Concomitantly, the establishment of favorable habitat for other microbial communities likely enhances these effects. Overall, over the short term, microalgae can functionally promote topsoil structural stability and thus provide protection against erosion in temperate agricultural soils. However, it is important to consider these dynamics in a broader perspective and examine the benefits of soil aggregation in relation to agricultural practices (e.g., soil tillage and agrochemical inputs), in order to promote the value of soil algae and cyanobacteria as soil conditioners or biofertilizer (Metting, 1990; Renuka et al., 2018).

The application of herbicides can change the microalgal communities and physicochemical parameters, which mean it can also change community functions. In particular, herbicides can disturb the growth of soil microalgae and thus alter their functional role in soil aggregate formation. This greenhouse study explored the impacts of an herbicide on soil aggregation. However, it is important to also look at community changes *in situ*, taking into account edaphic conditions (i.e., seasonal effects) and additional agricultural practices (i.e., soil tillage). More studies need to focus on soil photosynthetic communities, which are frequently overlooked in soil microbial eco(toxico)logy. Our findings highlight that specific functional groups (algae and cyanobacteria) or functional domain (microbiotic crusts) in soils can be promising bioindicators and original models for deciphering the impacts of agrochemical stress on soil function in agroecosystems.

## Author Contributions

OC and LC designed the study. LC, J-PP, and CM measured stability, and the microbial and biochemical parameters. J-PA analyzed the soil pigment composition. AB, SB, CL, and LT analyzed the soil exopolysaccharides. OC wrote the first draft of the manuscript with the help of AB, who contributed to the revision process, and minor comments were made by the other authors.

## Conflict of Interest Statement

The authors declare that the research was conducted in the absence of any commercial or financial relationships that could be construed as a potential conflict of interest.

## Footnotes

1 http://www.lille.inra.fr/las
