# Supplementary material for: Soil Photosynthetic Microbial Communities Mediate Aggregate Stability: Influence of Cropping Systems and Herbicide Use in an Agricultural Soil
Source: Front Microbiol. 2019 Jun 14;10:1319. doi: 10.3389/fmicb.2019.01319 (PMC6587365; doi:10.3389/fmicb.2019.01319)
Supplement: Supplementary file 1 [file Data_Sheet_1.PDF]

Figure S1

Figure S1: Principal Component Analysis of the photosynthetic pigments extracted from the soil aggregates, at the end of incubation, depending on the IPU treatment. on the Organic and Conventional soils. The contribution of each pigment to the ordination was shown by the corresponding vectors. Two electrophoresis pics can not be attributed to a given pigment according to our standard (n = 3).

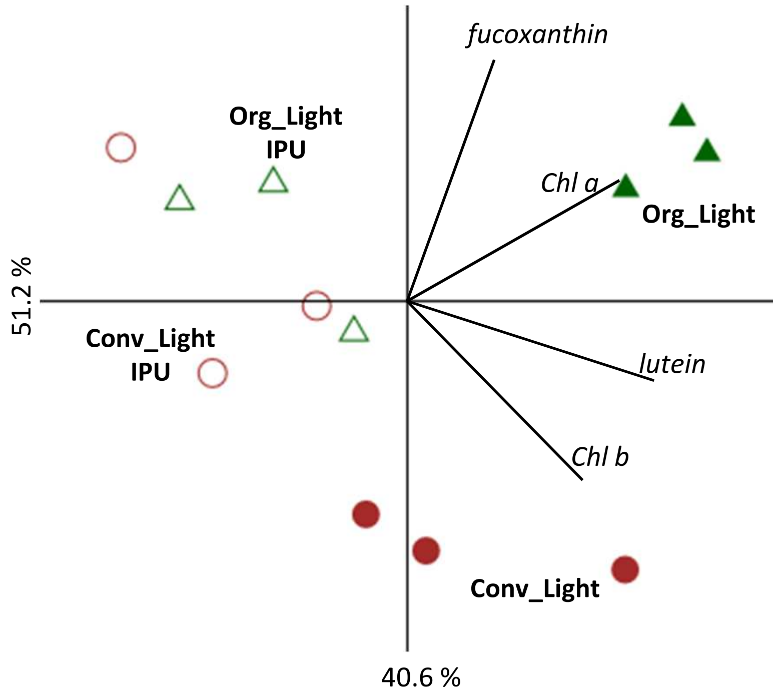

Figure S2

Figure S2: Monosaccharidic composition of bound carbohydrates-EPS samples (pool of triplicate EPS lyophilised samples) extracted from soil aggregates from Organic and Conventional cropping systems in soil microcosms at the end of the three incubation treatments (day 50). *Rha: rhamnose, Fuc: fucose, Ara: arabinose, Xyl: xylose, Man: mannose, Gal: galactose, Glc: glucose, GalA: galacturonic acid.*

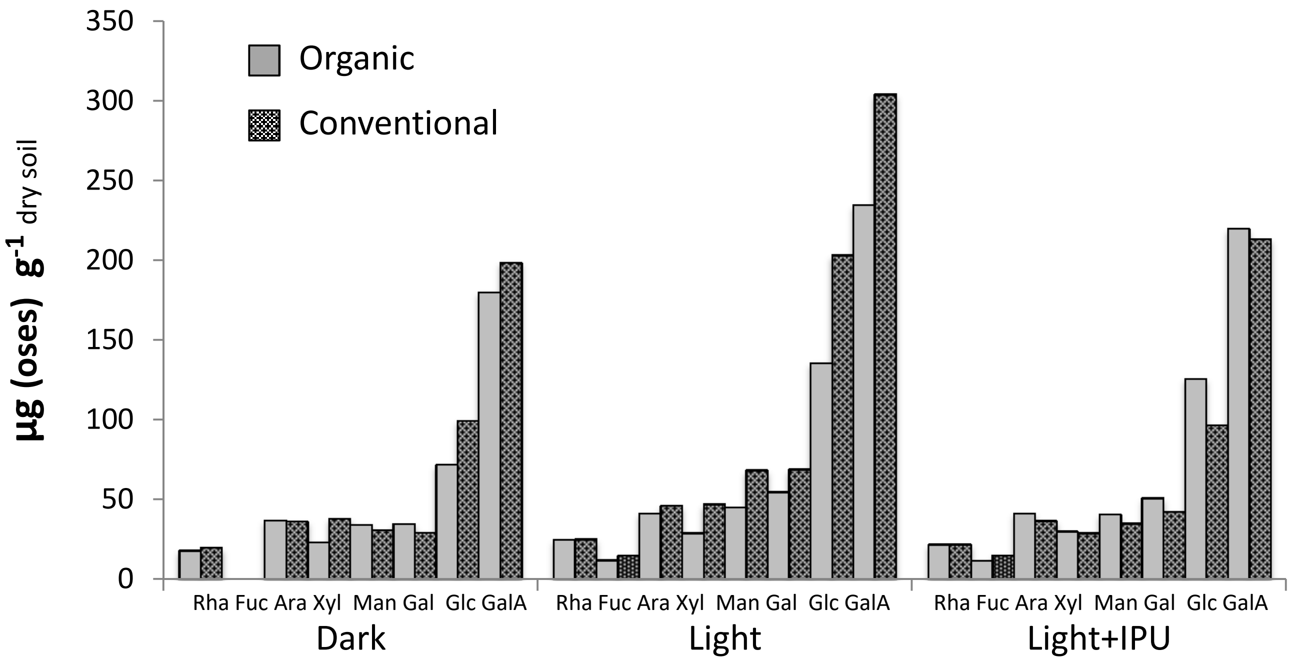

Figure S3

<u>Figure S3</u>: Correlations between physical (coarsest fragments > 2mm geometric mean between the three tests) and biological indicators of soil photosynthetic microbial communities (Chl a and FDA), total carbohydrate bound-EPS and C_{org}, in microcosms of the Organic (Green) and Conventional (Brown) soil.

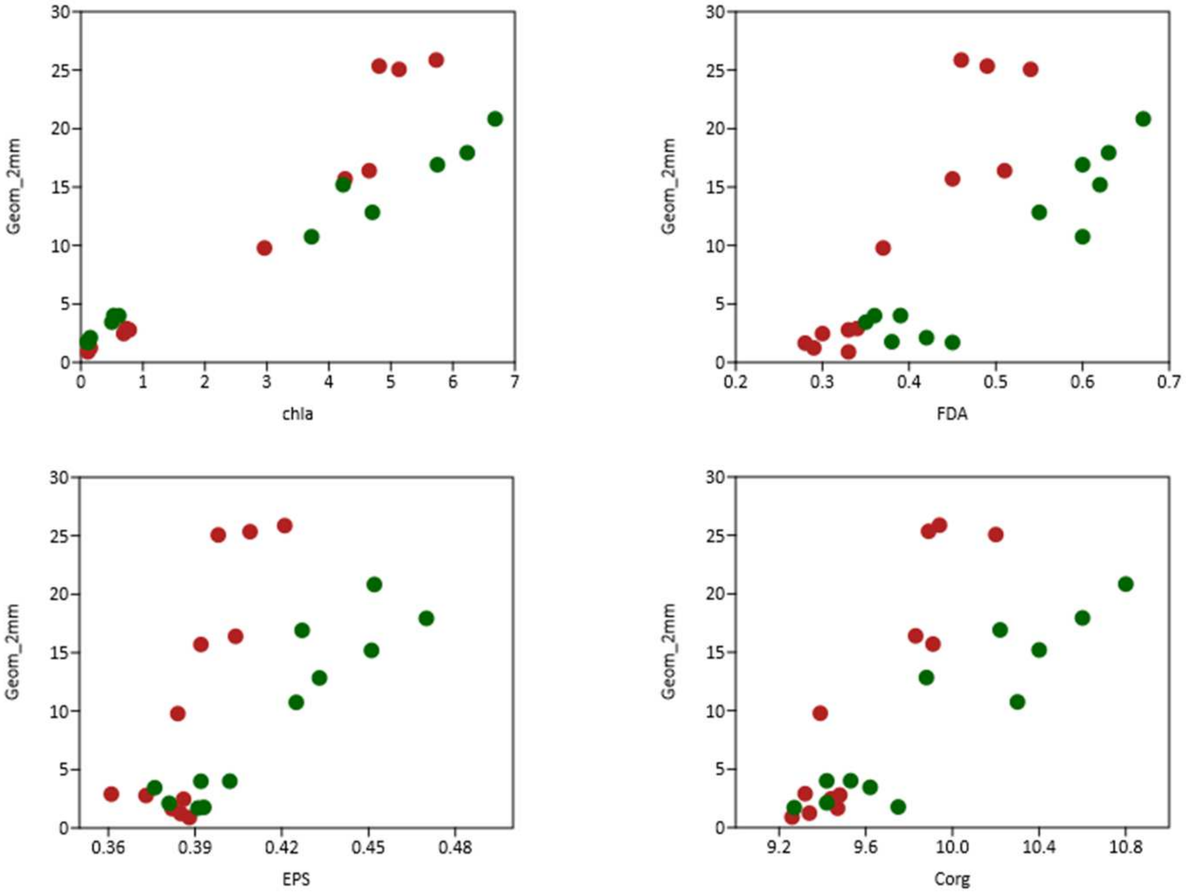

Table S1: Two-ways Permanova analyses of the aggregate class size distribution, following each disaggregation test.

Test_1	Df	SumOfSqs	R2	F	Pr(>F)
Treatment	3	0.191	0.434	59.4	0.001 ***
Crop.syst	1	0.213	0.485	199.3	0.001 ***
interaction	3	0.019	0.042	5.8	0.001 ***
Residual	16	0.017	0.039		
Total	23	0.440	1.000		

Test_2	Df	SumOfSqs	R2	F	Pr(>F)
treatment	3	0.27838	0.62778	49.5456	0.001 ***
Crop.syst	1	0.09793	0.22085	52.2899	0.001 ***
interaction	3	0.03715	0.08379	6.6127	0.001 ***
Residual	16	0.02997	0.06758		
Total	23	0.44343	1.00000		

Test_3	Df	SumOfSqs	R2	F	Pr(>F)
treatment	3	0.146387	0.57886	42.4156	0.001 ***
Crop.syst	1	0.064809	0.25628	56.3355	0.001 ***
interaction	3	0.023287	0.09208	6.7473	0.001 ***
Residual	16	0.018407	0.07279		
Total	23	0.252889	1.00000		
